# Postoperative Adjuvant Treatment in Women with Stage I Endometrial Cancer: A Retrospective Study

**DOI:** 10.1155/2023/4007616

**Published:** 2023-03-31

**Authors:** Peiying Fu, Haiying Sun, Ting Zhou, Pengfei Cui, Shixuan Wang, Ronghua Liu

**Affiliations:** Department of Obstetrics and Gynaecology, Tongji Hospital, Tongji Medical College, Huazhong University of Science and Technology, Wuhan, China

## Abstract

**Objective:**

To evaluate whether postoperative adjuvant treatment is beneficial for patient survival after surgery for early stage endometrial cancer (EC). We analyzed the outcomes of patients treated with radiotherapy, chemotherapy, or progestagen combined with other adjuvant treatments.

**Methods:**

We analyzed the outcomes of patients treated with radiotherapy alone, chemotherapy alone, or progestagen treatment with other adjuvant treatments. Women without any adjuvant treatment after operation were used as controls. We retrospectively examined disease-free survival (DFS), overall survival (OS), and high-risk factors that affected the survival status of all patients who received different postoperative adjuvant therapies.

**Results:**

In all 192 patients, the total relapse and mortality rates were 5.57% and 1.68%, respectively. Fourteen patients (7.29%) developed isolated local recurrence, and 2 patients died (1.04%) of recurrence during the follow-up period. The 5-year DFS and OS rates of all patients were 95.83% and 93.75%, respectively. No significant differences were observed in the 5-year DFS, 5-year OS, OS, or DFS among the four groups of patients with FIGO stage I endometrial cancer (*P*=0.9849, 0.7430, 0.9754, and 0.4534, respectively). The differences in the log-rank test results of the estimates of the 5-year DFS, 5-year OS, DFS, and OS of patients with different disease stages and different ages were all significant, but no differences were observed in these parameters among patients with varying degrees of differentiation. Histologic grade, CA125 level, ER and PR status, and adjuvant therapy had no significant effect on the DFS and OS of all patients according to univariate and multivariate regression analyses, but a significant effect on DFS and OS was found when the patients were stratified by age.

**Conclusion:**

This retrospective study showed that adjuvant therapy after surgery was not significantly associated with improved DFS or OS in patients with early stage endometrial cancer. However, FIGO stage and age affected the survival of patients with stage I endometrial cancer.

## 1. Introduction

In China, 63,400 new cases of endometrial cancer and 21,800 deaths from this cancer type were reported in 2015 [1]. Adjuvant therapy is regarded as the backbone of treatment for advanced endometrial cancer, but the optimal strategy to prevent recurrence and improve survival outcomes is still controversial, especially for early stage endometrial cancer. Therefore, the selection of appropriate postoperative adjuvant therapy for patients with early stage endometrial cancer is challenging.

Lymphovascular space invasion (LVSI) is considered to be an independent prognostic factor for survival and recurrence, and it has been shown to be related to lymphatic metastasis [2–4]. Patients with low-risk and low-intermediate-risk factors do not need adjuvant treatment, as they would not derive any benefit. Moreover, the optimal adjuvant treatment for patients with intermediate-risk and high-intermediate-risk factors is still controversial. In recent years, many doctors have reconsidered using chemotherapy for high-intermediate risk (HIR) stage I endometrial cancer despite the insufficiency of randomized data to support this. The results of a Cochrane Collaboration meta-analysis suggested a small numerical benefit in PFS and OS after patients received platinum-based chemotherapy for endometrial cancer [5]. However, some doctors have expressed different viewpoints. According to a systematic review, adjuvant chemoradiotherapy had no advantage over radiotherapy alone for overall survival and failure-free survival in high-risk patients with FIGO stages I-II endometrial cancer [6]. Therefore, whether early stage high-risk patients can benefit from adjuvant chemotherapy is worthy of further exploration.

The value of progestogenic agents in advanced endometrial carcinoma has been well demonstrated, while their role as adjuvant therapies in early stage endometrial cancer is somewhat contentious. Some studies have shown that adjuvant progestagen therapy can reduce recurrence and improve the survival rate of patients with early endometrial cancer. In the 1970s and 1980s, several small studies suggested a survival benefit from progestagen in patients with endometrial cancer [7]. However, in observational studies, progestagens have been demonstrated to have a limited role in preventing recurrence compared with control treatments [8]. Since then, researchers have paid little attention to adjuvant endocrine therapy for early stage endometrial cancer.

Therefore, the primary theme of this study was to retrospectively evaluate the oncologic outcomes and survival statuses associated with various postoperative adjuvant therapies and to assess the risk factors that affect the status of women with stage I endometrial cancer.

## 2. Materials and Methods

### 2.1. Patients

We retrospectively reviewed patients treated for endometrial cancer at our hospital (one of the major tertiary referral hospitals in China) from 2006 to 2016, and 654 patients diagnosed with FIGO stages I–IV endometrial cancer were identified. The Ethics Committee of Tongji Hospital, Tongji Medical College, Huazhong University of Science and Technology, approved this study (TJ-IRB20210737). After diagnosis, all patients underwent either laparoscopic, abdominal total hysterectomy, or bilateral salpingo-oophorectomy with or without pelvic/para-aortic lymph node dissection and pelvic washing. Two gynecologic pathologists reviewed and confirmed the pathologic specimens. All patients were diagnosed with stages I–IV endometrial cancer according to the revised FIGO staging criteria [9]. Surgical treatment was followed by adjuvant radiotherapy (RT) alone, chemotherapy (CT) alone, hormone therapy with other adjuvant treatments, or no further adjuvant treatment.

### 2.2. Adjuvant Therapy after Surgery

The selection of adjuvant therapy was at the discretion of the attending gynecological oncologist who managed the patient. Treatment of patients was performed according to international guidelines and included vaginal brachytherapy. The chemotherapy regimen used at our institution during the study period was platinum-based chemotherapy, with paclitaxel (135–175 mg/m^2^) and carboplatin (AUC = 5) given for two to four cycles every 3 weeks. Progestagen treatment was initiated 3-4 weeks after surgery or after other adjuvant treatments ended. The medroxyprogesterone acetate (MPA) dose was 250–500 mg administered once per day. This treatment was continued for 1 year.

### 2.3. Follow-Up

Follow-up examinations were performed at either our institution or at hospitals local to the patients. All patients were followed-up after the completion of comprehensive treatment every 3 months for the first 2 years, every 6 months during the next 3 years, and then yearly thereafter during the study period. The oncological status of patients at the last medical visit was also assessed and determined to be either remission, recurrence, or death.

### 2.4. Statistical Analysis

SPSS 20.0 statistical software was used for the statistical analysis; the “survival” package was utilized to apply complete survival analysis to the right-censored data, while the results were visualized with the “survminer” package. The univariate comparison of the four groups was summarized by descriptive statistics and tested by Fisher's exact method. The count data were expressed as *N* (%), and the chi-square test was used to analyze differences between groups. All data are represented as the mean ± standard deviation (SD), and variance analysis was used to test for differences between groups. The log-rank test was used for single-factor analysis of OS or DFS, and the Cox proportional risk model was used for multifactor analysis. The statistical significance threshold was *P*  <  0.05.

## 3. Results

### 3.1. Patients and Tumor Characteristics

**Figure 1:**
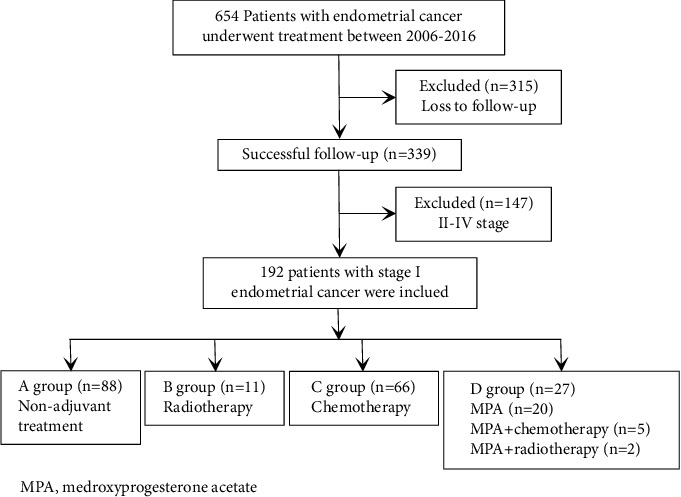
Retrospective study flow diagram.

In all stage of EC patients, 339 patients were successfully followed-up, and 315 patients were lost to follow-up after treatment. Overall, 147 patients with advanced-stage disease (II–IV) were excluded from this study. Finally, 192 patients were enrolled in the retrospective analysis. The women were then divided into four groups (A group (45.83%), nonadjuvant treatment; B group (5.73%), radiotherapy; C group (34.38%), chemotherapy; D group (14.06%), MPA alone or combined with chemotherapy or radiotherapy). Eleven patients and 66 patients received radiotherapy and chemotherapy, respectively. Twenty patients received adjuvant hormone therapy alone, and 7 patients received MPA combined with other adjuvant treatments (5 patients received chemotherapy and 2 patients received radiotherapy). The remaining 88 patients did not receive adjuvant treatment after surgery (Figure 1).

The baseline characteristics of all patients are summarized in Table 1. The median follow-up for all patients was 51 months (range: 18–143). No significant difference was found among the four groups in terms of age, histologic subtype, ER expression, or CA125 level in serum. A few patients were diagnosed with rare pathological types, such as adenocarcinoma (mesonephric-like), clear cell carcinoma, serous papillary carcinoma, mucinous papillary adenocarcinoma of the intestinal epithelium, and papillary adenocarcinoma (villous-tubular carcinoma subtype). The proportions of these rare subtypes did not differ among the groups. In addition, we found that histologic grade, FIGO stage, and PR expression differed among the four groups (*P*=0.0005, *P*=0.0112, and *P*=0.0064). Among 192 patients, 168 (87.5%) underwent pelvic lymphadenectomy, and 11.46% (22/192) underwent pelvic and para-aortic lymphadenectomy. No difference was found in the proportion of lymphadenectomy among the four groups (*P*=0.9688 and *P*=0.4211).

### 3.2. Survival Outcomes

**Figure 2:**
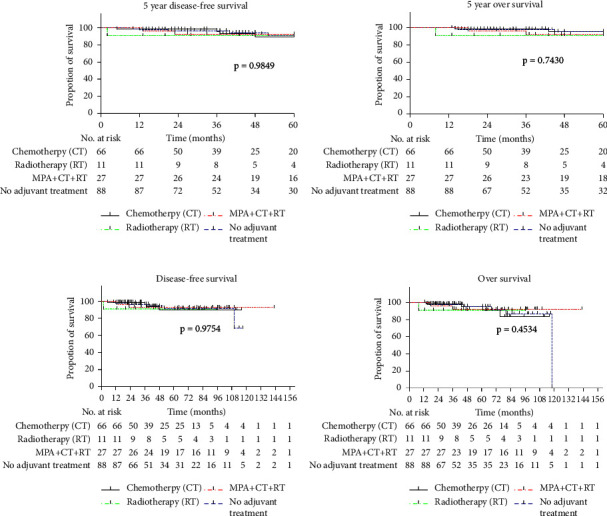
Kaplan-Meier estimates of the 5-year disease-specific survival, 5-year overall survival, overall survival and disease-specific survival among the four groups. Disease-free survival (a, c) and overall survival (b, d) after treatment with chemotherapy, radiotherapy or MPA + radiotherapy/chemotherapy. Patients who received no adjuvant treatment after surgery served as controls. The corresponding *P*-values are 0.9849, 0.7430, 0.9754 and 0.4534, respectively. Tick marks indicate censored data.

The total relapse and mortality rates of all 192 patients were 5.57% and 1.68%, respectively. During the follow-up period, 14 patients (7.29%) developed isolated local recurrence, and 2 patients (1.04%) died of recurrence. The 5-year OS and DFS rates of all patients were 93.75% and 95.83%, respectively. In this study, the log-rank test was used to test the significance of different treatments. No statistically significant differences were observed in the 5-year DFS, 5-year OS, OS, or DFS among the four groups of patients with stage I endometrial cancer (*P*=0.9849, 0.7430, 0.9754, and 0.4534) (Figure 2). This result suggested that adjuvant chemotherapy, radiotherapy, or hormone therapy + chemotherapy/radiotherapy after surgery did not improve the DFS or OS rates of patients with stage I endometrial cancer.

**Figure 3:**
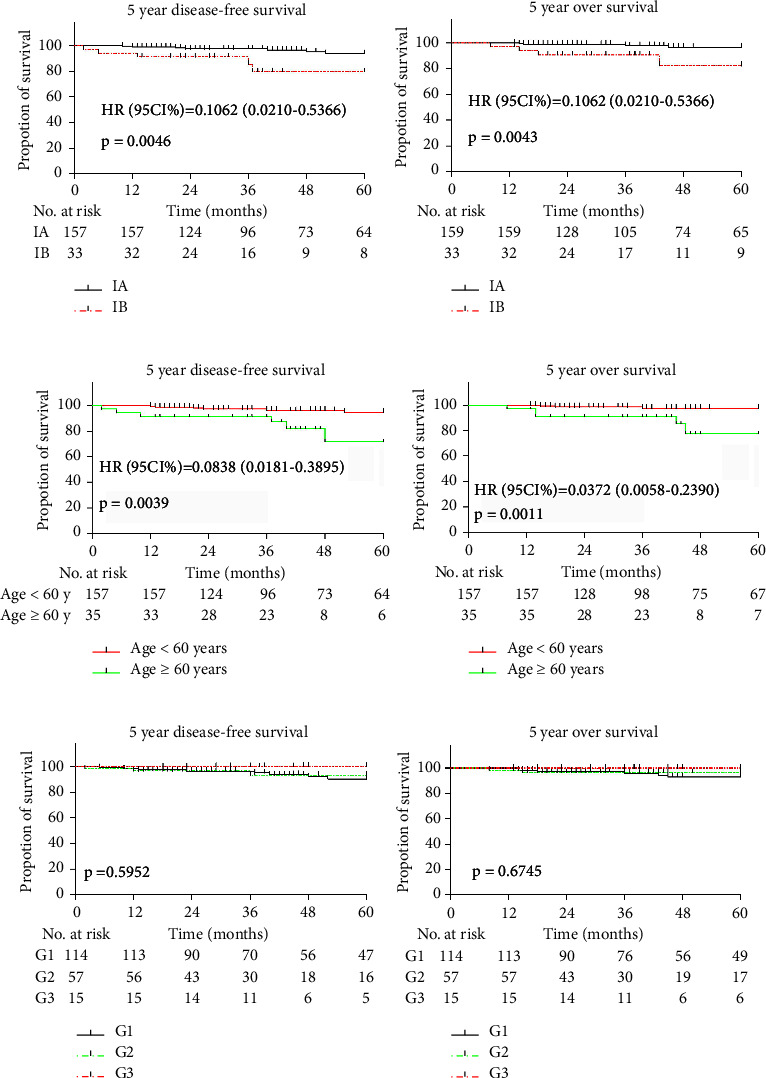
Estimates of the 5-year disease-free survival and 5-year overall survival in all patients by FIGO stage, age and degree of differentiation. Kaplan-Meier survival curves for the 5-year disease-free survival and 5-year overall survival by FIGO stage (a, b) and age (c, d). [(a), HR (95% CI): 0.1062 (0.0210–0.5366), *P*=0.0046; (b) HR (95% CI): 0.0566 (0.078–0.4047), *P*=0.0043; (c) HR (95% CI): 0.0838 (0.0181–0.3895), *P*=0.0039; and (d) HR (95% CI): 0.0372 (0.0058–0.2390), *P*=0.0011]. Panels (e) and (f) show disease-free survival and overall survival according to different degrees of differentiation, respectively (e)*P*=0.5952; (f)*P*=0.6475). Tick marks indicate censored data.

**Figure 4:**
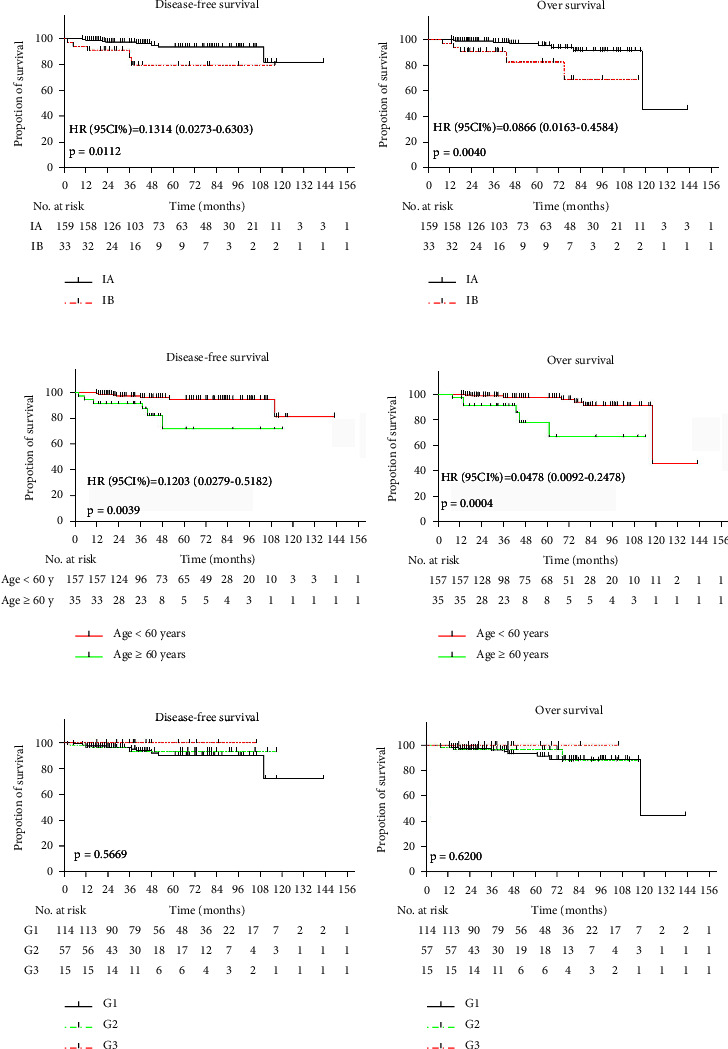
Disease-free survival and overall survival of all patients by FIGO stage, age and histologic grade. Kaplan-Meier survival curves for disease-free survival and overall survival by FIGO stage in all patients who received with different adjuvant treatments compared with patients who received no adjuvant treatment. Tick marks indicate censored data [(a), HR (95% CI): 0.1314 (0.0273–0.6303), *P*=0.0112; and (b) HR (95% CI): 0.0866 (0.0163–0.4584), *P*=0.0040]. DFS (c) and OS (d) by age [(c), HR (95% CI): 0.1203 (0.0279–0.5182), *P*=0.0040; and (d) HR (95% CI): 0.0478 (0.0092–0.2478), *P*=0.0004]. Panels (e) and (f) show the DFS and OS, respectively, according to different degrees of differentiation (e)*P*=0.5669; and (f)*P*=0.6200).

Not all types of postoperative adjuvant treatment are conducive to survival. Therefore, we analyzed the influence of stage, age, and tumor differentiation on the 5-year DFS, 5-year OS, DFS, and OS of early endometrial cancer patients. The differences in log-rank tests of the estimates of 5-year DFS, 5-year OS, DFS, and OS between stage IA and stage IB were all significant (hazard ratio, 0.1062, 95% CI, 0.0210–0.5366, *P*=0.0046; hazard ratio, 0.0566, 95% CI, 0.078–0.4047, *P*=0.0043; hazard ratio, 0.1062, 95% CI, 0.0273–0.6303, *P*=0.0112; and hazard ratio, 0.0866, 95% CI, 0.0163–0.4584, *P*=0.0040, respectively), which is similar to the pattern seen between different age groups in 5-year DFS, 5-year OS, DFS, and OS (hazard ratio, 0.0838, 95% CI, 0.0181–0.3895, *P*=0.0039; hazard ratio, 0.0372, 95% CI, 0.0058–0.2390, *P*=0.0011; hazard ratio, 0.1203, 95% CI, 0.0279–0.5182, *P*=0.0040; and hazard ratio, 0.0478, 95% CI, 0.0092–0.2478, *P*=0.0004, respectively). It is worth mentioning that the 5-year DFS, 5-year OS, DFS, and OS rates were not different between the different groups stratified by tumor differentiation status (*P*=0.5952, 0.6475, 0.5669, and 0.6200) (Figures 3 and 4).

### 3.3. The Clinical Factors That Affect Survival Status

To assess the risk factors that affect the survival status of women with stage I endometrial cancer, we performed a univariate analysis of different variables. The log-rank test (time series test) was used for the univariate analysis to analyze the impact of various factors on prognosis. Histologic grade, estrogen receptor status, progesterone receptor status, and CA125 level were not associated with significant differences in DFS and OS (log-rank, *P*=0.6946, 0.9199, 0.9347, and 0.3272), but patient age was associated with prognosis (log-rank, *P*=0.0045 for DFS and 0.0003 for OS) (Table 2).

Furthermore, a Cox proportional hazard model was used for the multivariate regression analysis to determine the risk factors that affect the DFS or OS of stage I endometrial cancer patients. Six factors (age, histologic grade, ER status, PR status, CA125 level, and adjuvant treatment) were introduced into the Cox model as independent variables. At a threshold of *P*  <  0.05, no significant difference was found in prognosis among different histologic grades, ER and PR statuses, CA125 level, and adjuvant treatment status (Table 3). However, age affected DFS and OS in early-stage endometrial cancer (HR (95% CI): 6.119 (1.502–24.924), *P*=0.0115; and HR (95% CI): 9.088 (2.012–41.058), *P*=0.0041).

## 4. Discussion

As China has become an aging society, the number of women who experience obesity and the incidence of EC have proportionally increased. EC has the strongest link with obesity, every 5 kg/m^2^ increase in BMI associated with 54% increase in EC risk [10]. Obesity creates a proinflammatory milieu, which might contribute to endometrial cancer risk [11]. Approximately 80% of endometrial cancers are hormone receptor-positive endometrioid adenocarcinomas. Most endometrioid carcinomas are well to moderately differentiated [12]. The percentages of patients with G1-G2 and G3 were 89% and 11%, respectively. The clinical data of this study are consistent with the literature and suggest that approximately 10% of endometrial cancers are type-2 (high-grade) lesions. Up to 40% of nonendometrioid endometrial cancers are mixed with an endometrioid component [13]. In our study, a few cases were diagnosed with rare pathological types, such as the endocervical type, clear cell carcinoma, mucinous papillary adenocarcinoma of the intestinal epithelium, the villous-tubular carcinoma subtype, and serous papillary carcinoma. Since the number of cases was small, it was difficult to evaluate the prognosis of these subtypes and their impact on the results of this study.

Whether adjuvant therapy should be administered after surgery for early endometrial cancer is still controversial. NCCN guidelines recommend that complementary radiotherapy or systemic therapy be considered if patients have potential high-risk factors, including age ≥60 years, deep myometrial invasion, and/or LVSI [14]. Patients with focal or substantial LVSI received different adjuvant treatments according to a three-tiered system that quantitates LVSI. In contrast, the presence of LVSI is associated with a high risk of mortality in patients with early stage well-differentiated endometrial carcinoma [15]. The Cancer Genome Atlas (TCGA)-typing for EC was proved as a tool for guiding treatment. But the data of TCGA typing in this retrospective study were missing, and we could not analyze the factor in our study. No difference was found in the survival rates between groups after a 5-year follow-up [16]. The current retrospective study revealed no statistically significant differences in the 5-year DFS, 5-year OS, OS, or DFS across the four groups of stage I endometrial cancer patients. Similar results were observed in two randomized trials (GOG-99 and PORTEC-1) and a retrospective study, which suggested no overall survival advantage of adjuvant radiation in patients with stage I, high-intermediate risk cancer [17–19]. In contrast, 50% of the 192 patients presented to our hospital for secondary surgery or further follow-up treatment after their first surgery, which was performed at other hospitals. As the LVSI status of these patients was unknown, we could not evaluate this factor.

In a few studies, researchers reported that adjuvant endocrine therapy may provide a benefit in terms of delaying recurrence or prolonging the survival of patients with early endometrial cancer [20]. However, most randomized trials of adjuvant progestagen therapy have failed to show any advantage in endometrial cancer, and data have even revealed that death due to cardiovascular disease tended to be higher in the progestagen group than in the control group [21–23]. These findings are consistent with our results. In our study, carboplatin plus paclitaxel was adopted as postoperative adjuvant chemotherapy in many patients with stage I disease. The data suggested that women gain little benefit from adjuvant chemotherapy, including women with high-risk factors, such as deep myometrial invasion and the serous or clear cell histologic type. Indeed, many studies have demonstrated that adjuvant chemotherapy for high-risk endometrial cancer does not improve survival rates [24, 25]. However, the results of randomized trials have varied, and some previous studies have suggested that adjuvant chemotherapy after surgery is beneficial for early stage EC with HIR factors [26]. Furthermore, two retrospective studies showed that adjuvant platinum-based chemotherapy plus vaginal brachytherapy (VBT) achieved excellent results in high-risk early-stage endometrial cancer [27, 28]. However, we were unable to confirm this result because this retrospective study contained no such cases.

According to our retrospective observations, the differences in DFS and OS were significant between stage IA and stage IB. That is, even in early endometrial cancer, the FIGO stage still affects the DFS and OS of endometrial cancer patients. Moreover, age (<60 years and ≥60 years) was another factor influencing the prognosis of patients with early stage endometrial cancer. Notably, according to both the univariate and multivariate analyses, ER status, PR status, CA125 level, histologic grade, and the type of adjuvant therapy did not affect PFS or OS in FIGO stage I endometrial cancer patients in our study, but age was associated with differences in OS and PFS. Many physicians have verified that the cancer antigen 125 (CA125) level, age older than 60 years, and depth of myometrial invasion >50% were significant factors for overall survival in a retrospective study [29]. Age >60 years (or > 50 years) and degree of differentiation may be high-intermediate risk factors according to a systematic review of guidelines in the US [30]. Among all the factors analyzed in our study, age was the only indicator that independently affected OS and DFS in stage I endometrial cancer. Due to incomplete data on lymph node metastasis in this study, it was not possible to analyze this factor and its impact on survival status.

The goal of adjuvant therapy in endometrial cancer is to reduce the risk of disease recurrence, and whether postoperative adjuvant therapy should be used for early endometrial cancer is controversial. Our results agree with the above findings and suggest that postoperative adjuvant treatments are not associated with better OS or DFS in patients with either FIGO stage IA or stage IB endometrial cancer. All the above evidence seems to support the conclusion that women do not gain a survival advantage from postoperative adjuvant therapy after surgery for stage I endometrial cancer regardless of disease stage (IA and IB). Nevertheless, our study still has some shortcomings. The main limitation of our study is that we included in our analysis with retrospective study nature. It is limited by its retrospective nature, the heterogeneity of the data and the reliance on clinical endometrial cancer data not originally collected for research purposes. We could only analyze the results from the follow-up data as the lack of some clinical data, such as LVSI and a high rate of loss to clinical follow-up. Additionally, it was limited by single-center experience and potential selection bias, which may limit its external validity. Our results may not represent the findings of other hospitals.

Therefore, it is necessary to further discuss the benefits and disadvantages of adjuvant endocrine therapy, chemotherapy, and radiotherapy as well as the major risk factors for early endometrial cancer. In the future, it will be possible to achieve the goal of personalized treatment for individual patients.

## Figures and Tables

**Table 1:** Patient and tumor characteristics.

Characteristic	A group	B group	C group	D group	*P*-value
*N*	88	11	66	27	
Age (%)					0.2823
<60 y	69 (78.41)	7 (63.64)	55 (83.33)	24 (88.89)	
≥60 y	19 (21.59)	4 (36.36)	11 (16.67)	3 (11.11)	
Histologic type (%)					0.7971
Adenocarcinoma	81 (92.04)	11 (100)	62 (93.93)	27 (100)	
Adenocarcinoma (mesonephric-like)	3 (3.41)	0 (0)	0 (0)	0 (0)	
Clear cell carcinoma	1 (1.14)	0 (0)	3 (4.55)	0 (0)	
Mucinous papillary (adenocarcinoma of intestinal epithelium)	1 (1.14)	0 (0)	0 (0)	0 (0)	
Papillary adenocarcinoma (villous-tubular carcinoma subtype)	0 (0)	0 (0)	1 (1.52)	0 (0)	
Serous papillary carcinoma	2 (2.27)	0 (0)	0 (0)	0 (0)	
Histologic grade (%)					0.0005^*∗*^
G1	64 (72.73)	4 (36.36)	26 (39.40)	20 (74.07)	
G2	17 (1932)	5 (4545)	30 (4545)	5 (1852)	
G3	3 (3.41)	2 (18.18)	8 (12.12)	2 (7.41)	
Unknown	4 (4.54)	0 (0)	2 (3.03)	0 (0)	
FIGO stage (%)					0.0112^*∗*^
IA	80 (90.91)	7 (63.64)	49 (74.24)	23 (85.19)	
IB	8 (9.09)	4 (36.36)	17 (25.76)	4 (14.81)	
Lymphadenectomy					
Pelvic lymph node	62 (70.45)	9 (81.82)	54 (81.82)	21 (77.78)	0.9688
Pelviclymph + para-aortic node	12 (12.5)	1 (0.91)	8 (12.12)	1 (3.7)	0.4211
CA125					0.395
<35 U/ml	40 (45.45)	6 (54.55)	35 (53.03)	10 (37.04)	
≥35 U/ml	11 (12.50)	2 (18.18)	6 (9.09)	1 (3.70)	
Unknown	37 (42.05)	3 (27.27)	25 (37.88)	16 (59.26)	
ER (%)					0.0743
+	48 (54.55)	10 (90.91)	49 (74.24)	17 (62.97)	
−	8 (9.09)	0 (0)	6 (9.09)	1 (3.70)	
Unknown	32 (36.36)	1 (9.09)	11 (16.67)	9 (33.33)	
PR (%)					0.0064^*∗*^
+	41 (46.59)	7 (63.64)	45 (68.18)	18 (66.67)	
−	15 (17.05)	3 (27.27)	9 (13.64)	0 (0)	
±	0 (0)	0 (0)	1 (1.52)	0 (0)	
Unknown	32 (36.36)	1 (9.09)	11 (16.66)	9 (33.33)	

A group, no adjuvant treatment; B group: radiotherapy; C group, chemotherapy; D group, MPA + chemotherapy (or) radiotherapy. Abbreviations: FIGO, international federation of gynecology and obstetrics; CA125, carbohydrate antigen-125; ER, estrogen receptor; PR, progesterone receptor. ^*∗*^*p* < 0.05.

**Table 2:** Univariate analysis for risk factors affecting the survival status of women with stage I endometrial cancer.

Parameter	Disease-specific survival		Overall survival	
	Chi-square	*P*-value	Chi-square	*P*-value
Age	8.0783	0.0045^*∗*^	13.1273	0.0003^*∗*^
Histologic grade	1.5171	0.6783	1.4468	0.6946
ER status	0.6729	0.7143	0.1670	0.9199
PR status	0.0054	0.9973	0.1350	0.9347
CA125	1.1792	0.2775	0.9601	0.3272
Adjuvant treatment	0.2145	0.9752	0.4775	0.9238

^*∗*^ *p* < 0.05

**Table 3:** Multivariate analysis of risk factors affecting the disease-specific survival and overall survival status in women with stage I endometrial cancer.

Parameter	Disease-specific survival		Overall survival	
	HR (95% CI)	*P*-value	HR (95% CI)	*P*-value
Age	6.119 (1.502–24.924)	0.0115^*∗*^	9.088 (2.012–41.058)	0.0041^*∗*^
Histologic grade	0.335 (0.079–1.412)	0.1361	0.49 (0.11–2.186)	0.3501
ER	0.133 (0.009–1.913)	0.138	0.227 (0.011–4.551)	0.3325
PR	0.974 (0.062–15.207)	0.9848	1.366 (0.063–29.5)	0.8424
CA125	—	0.9952	—	0.9961
Adjuvant	1.513 (0.769–2.976)	0.2302	1.651 (0.828–3.294)	0.1547

^*∗*^ *p* < 0.05

## Data Availability

The data used to support the findings of this study are included within the article.
